# Branch Retinal Vein Occlusion Revealing Previously Undiagnosed Systemic Disease

**DOI:** 10.7759/cureus.98675

**Published:** 2025-12-07

**Authors:** Daria Valipur Kolti, Jakub Juszczyk, Sara Omidi, Julia Kierner, Dariusz Valipur Kolti

**Affiliations:** 1 Medicine, University Clinical Centre of the Medical University of Warsaw, Warsaw, POL

**Keywords:** anti-vegf therapy, arterial hypertension, branch retinal vein occlusion, multidisciplinary care, retinal vascular disease

## Abstract

Branch retinal vein occlusion (BRVO) is a common cause of vision loss and frequently reflects an underlying systemic vascular disorder. Early systemic assessment is fundamental for preventing ocular and cardiovascular complications. This report describes the clinical case of a 61-year-old woman who presented with sudden, painless visual field loss in the left eye. Fundoscopic and angiographic findings confirmed superotemporal BRVO with macular edema. Intravitreal anti-vascular endothelial growth factor (VEGF) therapy was initiated, and referral to a primary care physician for systemic evaluation was requested. However, due to the absence of a standardized referral protocol, the patient did not undergo prompt assessment. One week after the initial ophthalmologic visit, she presented to the emergency department with a severe headache and was found to have a hypertensive crisis. This case focuses attention on how retinal vascular occlusion (RVO) can be the first manifestation of an undetected systemic hypertension. It also marks the gaps in current systemic screening protocols for RVO and the need for closer collaboration between ophthalmologists and internal medicine specialists. A comprehensive and multidisciplinary evaluation of BRVO is indispensable for uncovering systemic risk factors and guiding treatment. Timely ocular therapy combined with systemic management can prevent irreversible visual loss and systemic complications.

## Introduction

Retinal vein occlusion (RVO) is one of the principal retinal vascular disorders, representing a significant cause of visual impairment [1]. It is estimated to affect approximately 16 million people worldwide. Among its subtypes, branch retinal vein occlusion (BRVO) occurs predominantly among all ethnic groups [2]. The arterio-venous crossing anatomy is a key element of BRVO pathogenesis. An anteriorly running arteriole compresses the underlying vein, contributing to occlusion. As a consequence, patients often notice a subtle blurring in a part of their visual field. BRVO typically presents with characteristic fundoscopic image, such as intraretinal hemorrhages, dilated and tortuous veins, cotton-wool spots, and macular edema [3]. The multifactorial nature of RVO has been reflected in a case-control study by Rath et al. (1992), which identified systemic hypertension, open-angle glaucoma, and male sex as major independent risk factors [4]. Arterial hypertension remains the strongest risk factor in both subtypes [5]. Notably, hypertension and diabetes-related end-organ damage promote vascular remodeling, atherosclerotic changes, and impaired endothelial function, all of which markedly predispose individuals to BRVO [6]. Given the multifactorial pathogenesis of this condition and its implications beyond the eye, it is crucial to emphasize the role of ophthalmologists in early diagnosis and timely referral for systemic evaluation. The present case report highlights the importance of comprehensive care in BRVO in unmasking a previously undiagnosed systemic disease.

## Case presentation

Patient information 

A 61-year-old woman presented to the ophthalmology outpatient department with a sudden, painless narrowing of the visual field in the left eye, which had developed the day before. She denied any other accompanying symptoms, including pain, flashes, or floaters, and reported no systemic symptoms suggestive of underlying disease. The patient had no significant past medical history and was not taking any regular medications. Family history was notable for myocardial infarction and ischemic stroke. 

Ophthalmologic examination

**Figure 1:**
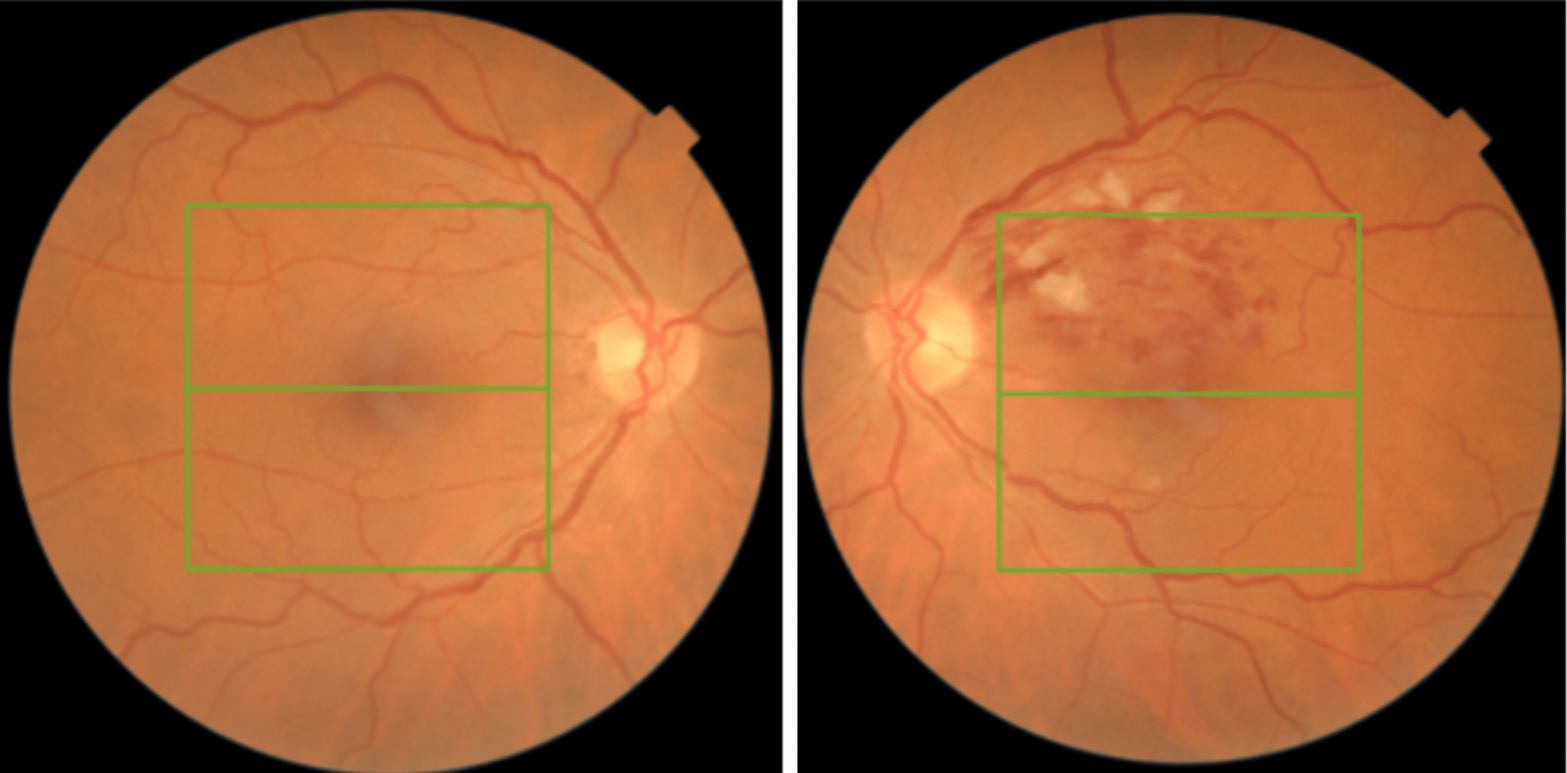
Fundus imaging of the right eye (left image) and the left eye (right image) The left eye demonstrates changes consistent with branch retinal vein occlusion.

On examination, her best-corrected visual acuity (BCVA) was 20/40 in the left eye, 20/30 in the right eye, and intraocular pressure was 15 mmHg bilaterally. The slit-lamp examination of the anterior segment was unremarkable in both eyes. Fundoscopic examination of the left eye revealed dilated, tortuous retinal veins, scattered retinal flame-shaped hemorrhages, retinal edema, and cotton wool spots, predominantly in the superior temporal quadrant. No abnormalities were observed in the right eye (Figure 1).

Imaging findings

**Figure 2:**
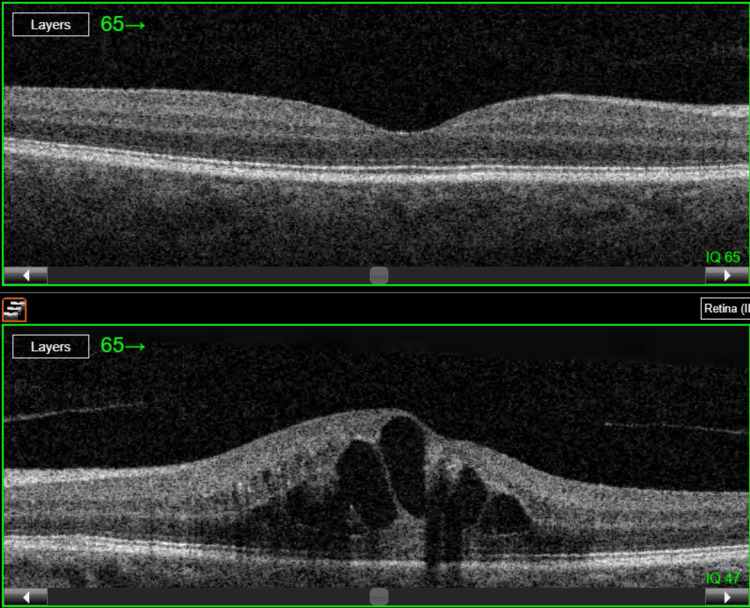
Optical coherence tomography (OCT) images of the right (top) and left (bottom) eye The scan of the left eye reveals cystoid macular edema associated with branch retinal vein occlusion.

Optical coherence tomography (OCT) of the macula showed retinal elevation with cystoid macular edema with a central retinal thickness (CRT) of 638 µm in the left eye, while OCT of the right eye visualized well-preserved retinal architecture, with a CRT of 173 µm (Figure 2). 

**Figure 3:**
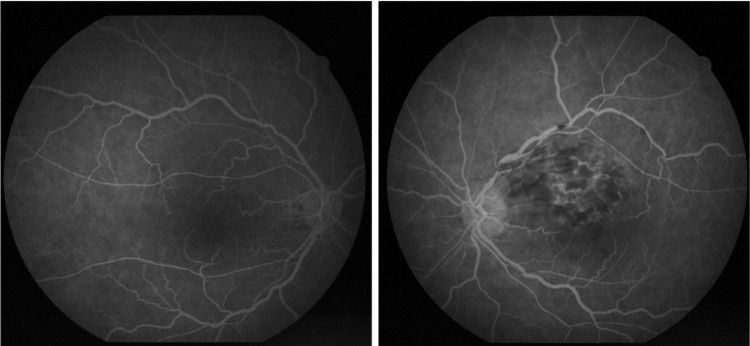
Fluorescein angiography of the right eye (left image) and the left eye (right image), demonstrating findings consistent with a superior temporal branch retinal vein occlusion in the left eye The affected venous branch appears dilated and tortuous, with venous anastomoses temporal to the macula. In the area of occlusion, dot-blot and flame-shaped hemorrhages, cotton wool spots, and retinal edema extending towards the macula are observed. The peripheral retina shows no evidence of ischemic areas.

Fluorescein angiography (FFA) confirmed non-ischemic BRVO in the superotemporal region, demonstrating delayed venous filling, venous leakage, and formation of collateral vessels. The retinal periphery showed no evidence of capillary non-perfusion or disturbed flow. The optic disc appeared normal (Figure 3).

Treatment and systemic evaluation

Based on these findings, the patient was referred to a primary care physician for an evaluation of possible systemic causes of BRVO, and treatment with intraocular anti-VEGF injections was proposed. Anti-VEGF therapy was chosen to reduce macular edema and improve visual acuity, as recommended by current clinical guidelines [7,8]. Additionally, prior to the anti-VEGF injections, the patient was prescribed sulodexide to improve retinal microcirculation and reduce the risk of further vascular events. Although its direct effect on visual outcomes in BRVO is not clearly proven, and its use varies between clinicians, sulodexide was administered as an adjunct due to its vascular-protective properties [9].

One week after the initial ophthalmologic evaluation, the patient woke up with a severe headache and presented to the emergency department, where her blood pressure was found to be 270/130 mmHg (normal value: 120/80 mmHg). Laboratory tests, including complete blood count, lipid profile, metabolic panel and blood glucose were performed. All results were within normal limits, ruling out alternative systemic causes, such as uncontrolled diabetes, hypercoagulable states, and inflammatory conditions (Table 1).

**Table 1:** Systemic and laboratory evaluation

Parameter	Result
Blood pressure	270/130 mmHg
Fasting blood glucose	Normal
HbA1c	Normal
Lipid profile	Normal
Complete blood count	Normal
Renal function/Electrolytes	Normal
Inflammatory markers	Normal

The patient was diagnosed with primary arterial hypertension and, after blood pressure stabilization, was started on an antihypertensive therapy including a calcium channel blocker (amlodipine) and a thiazide diuretic (hydrochlorothiazide) under cardiology supervision. 

Follow up

**Figure 4:**
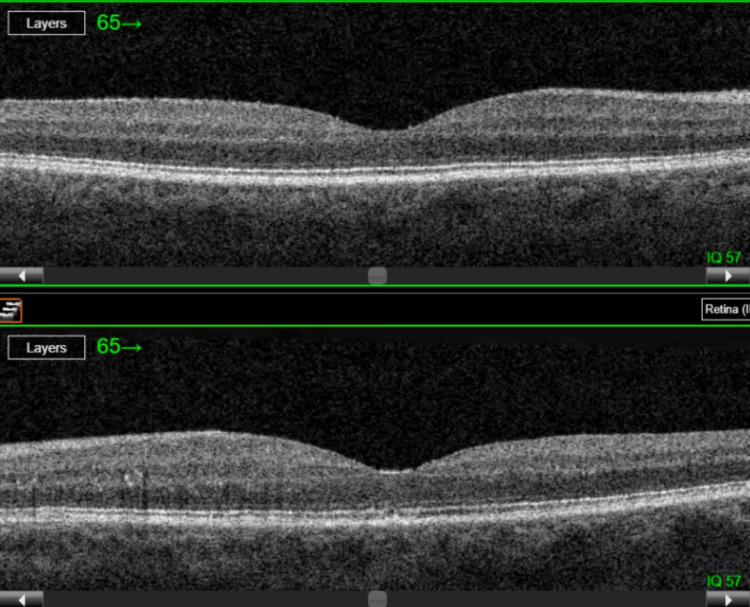
Follow-up optical coherence tomography (OCT) scans of the right eye (top) and left eye (bottom), 20 days after two intravitreal anti-VEGF injections into the left eye, showing complete resolution of macular edema in the left eye

Subsequent ophthalmology follow-up, 20 days after the first presentation, showed improvement in visual acuity after the first anti-VEGF injection and stabilization after the second. Currently further treatment is ongoing, with the following results in the left eye: BCVA 20/25 and CRT of 194 µm (Figure 4 and Table 2).

**Table 2:** Timeline of treatment and visual/anatomical outcomes in the left eye BCVA: Best Corrected Visual Acuity; VEGF: Vascular Endothelial Growth Factor.

Timepoint	Treatment	BCVA (Snellen)	Central retinal thickness (µm)	Notes
Baseline	—	20/40	638	Cystoid macular edema
After 1st Injection	Anti-VEGF	20/30	300	Improvement in macular edema
After 2nd Injection	Anti-VEGF	20/25	194	Complete resolution of macular edema
Ongoing follow-up	Anti-VEGF	20/25	194	Monitoring continued

## Discussion

In addition to being a retinal vascular disorder, BRVO often indicates an underlying systemic vascular malfunction, making it a major clinical issue in ophthalmology. Maintaining awareness of the close clinical correlation between BRVO and systemic vascular conditions is crucial for accurate evaluation and prevention of further complications. 

Hyperlipidemia, arterial hypertension, diabetes mellitus, and hormonal variables are commonly associated with BRVO. The pathophysiological connection between BRVO and systemic hypertension extends beyond compression of the retinal vein by a sclerotic artery at the arteriovenous crossing. Subclinical processes, such as turbulent blood flow, decreased venous outflow, and endothelial damage, create conditions that favor the formation of a parietal thrombus, often making visual symptoms the first indication of systemic disease [10].

A recent systematic review of clinical practice guidelines for the diagnosis and management of retinal vein occlusion revealed inconsistencies regarding recommendations for systemic screening. The majority of the reviewed guidelines, scored low in methodological rigor, particularly when it came to protocols for cardiovascular screening and hypertension management. These results suggest that current guidelines may not provide sufficiently detailed approaches to systemic risk evaluation, emphasizing the need for a more multidisciplinary model of care for patients with BRVO [7]. 

In the present case, despite prompt referral for systemic assessment, the lack of standardized screening guidance and delayed follow-up led to a late recognition of the underlying hypertension. This marks the clinical importance of implementing standardized systemic assessment protocols in patients with retinal vascular occlusions. Establishing clear and time-bound pathways for cardiovascular assessment could facilitate earlier detection of hidden hypertension and reduce the risk of preventable ocular and systemic complications. 

Other studies published in the literature have outlined retinal vascular occlusions as the first manifestation of systemic vascular disease. For example, Zhang et al. (2025) described a patient presenting with both BRVO and contralateral branch retinal artery occlusion (BRAO), who was found to have significant carotid atherosclerosis, suggesting a shared systemic pathology [11]. Considering the systemic vascular connections of BRVO, the local retinal pathology reflects the consequences of vascular compromise within the eye. A series of events, including disruption of the blood-retinal barrier, increased vascular permeability, and the development of macular edema are initiated. Moreover, sustained hypoxia promotes VEGF production, leading to neovascularization that may progress to vitreous hemorrhage or neovascular glaucoma if left untreated [12].

In this study, intravitreal anti-VEGF therapy significantly reduced macular edema and stabilized visual acuity, confirming the efficacy of this targeted treatment. Ocular complications, including macular edema, vitreous hemorrhage, and tractional retinal detachment, should be addressed promptly, with particular attention to resolving macular edema before irreversible damage occurs to the foveal photoreceptors, which are critical for central vision [13]. 

Given the association between retinal vein occlusions and potentially fatal events, a multidisciplinary approach is essential. Therefore, management should be integral not only for recognizing underlying systemic conditions such as hypertension, diabetes, dyslipidemia, or thrombophilic disorders but also for directing further ocular management. This shows that BRVO care should also be tailored to its underlying cause, since effective treatment requires addressing both ocular pathology and the systemic conditions that contribute to venous occlusion. 

This case underscores the importance of standardized systemic screening after retinal vascular occlusions and shows how delayed diagnosis of hypertension might accelerate disease progression. It acts as an educational reminder of the need for organized cooperation between ophthalmologists and internal medicine caregivers.

## Conclusions

BRVO represents a condition at the nexus of ophthalmology and internal medicine, where ocular findings may reveal hidden systemic disease. Prompt ocular care and systemic assessment may not only improve visual outcomes but also avert future cardiovascular complications. This case report illustrates how systemic hypertension may remain clinically silent until ocular vascular occlusion occurs, reinforcing the diagnostic relevance of retinal findings in the detection of systemic illness. The need for standardized systemic screening protocols and greater collaboration between ophthalmologists and internists to prevent irreversible vision loss and improve overall patient outcomes is strongly emphasized. 
